# Advances in the Preclinical Study of Some Flavonoids as Potential Antidepressant Agents

**DOI:** 10.1155/2018/2963565

**Published:** 2018-02-01

**Authors:** León Jesús German-Ponciano, Gilberto Uriel Rosas-Sánchez, Eduardo Rivadeneyra-Domínguez, Juan Francisco Rodríguez-Landa

**Affiliations:** ^1^Programa de Doctorado en Neuroetología, Instituto de Neuroetología, Universidad Veracruzana, Xalapa, VER, Mexico; ^2^Facultad de Química Farmacéutica Biológica, Universidad Veracruzana, Xalapa, VER, Mexico; ^3^Laboratorio de Neurofarmacología, Instituto de Neuroetología, Universidad Veracruzana, Xalapa, VER, Mexico

## Abstract

Flavonoids are phenolic compounds found commonly in plants that protect them against the negative effects of environmental insults. These secondary metabolites have been widely studied in preclinical research because of their biological effects, particularly as antioxidant agents. Diverse flavonoids have been studied to explore their potential therapeutic effects in the treatment of disorders of the central nervous system, including anxiety and depression. The present review discusses advances in the study of some flavonoids as potential antidepressant agents. We describe their behavioral, physiological, and neurochemical effects and the apparent mechanism of action of their preclinical antidepressant-like effects. Natural flavonoids produce antidepressant-like effects in validated behavioral models of depression. The mechanism of action of these effects includes the activation of serotonergic, dopaminergic, noradrenergic, and *γ*-aminobutyric acid-ergic neurotransmitter systems and an increase in the production of neural factors, including brain-derived neurotrophic factor and nerve growth factor. Additionally, alterations in the function of tropomyosin receptor kinase B and activity of the enzyme monoamine oxidase A have been reported. In conclusion, preclinical research supports the potential antidepressant effects of some natural flavonoids, which opens new possibilities of evaluating these substances to develop complementary therapeutic alternatives that could ameliorate symptoms of depressive disorders in humans.

## 1. Introduction

Depression is one of the most frequently diagnosed psychiatric disorders in the general population, the symptoms of which negatively impact health and are associated with high financial costs [1]. According to the World Health Organization, depression will become the primary cause of disability by 2030 [2].

A wide variety of antidepressant drugs are available to treat the symptoms of depression. Such antidepressants produce their therapeutic effects through actions on diverse neurotransmitter systems, including the serotonergic, noradrenergic, and dopaminergic systems [3]. The principal antidepressant drugs are tricyclic antidepressants (e.g., clomipramine and imipramine), monoamine oxidase inhibitors (e.g., phenelzine and selegiline), selective serotonin reuptake inhibitors (e.g., fluoxetine and fluvoxamine), selective dopamine reuptake inhibitors (e.g., amineptine and methylphenidate), selective norepinephrine reuptake inhibitors (e.g., reboxetine and viloxazine), and dual antidepressant drugs (e.g., venlafaxine and duloxetine) [4, 5].

Most antidepressant drugs have a delayed onset of therapeutic actions and many have side effects when taken in the long term. This has led patients to search for alternatives, based on the use of plants with reputed antidepressant activity [6]. An increasing number of studies have investigated natural chemical compounds with potential antidepressant activity [7], including bioactive metabolites, such as flavonoids, that exert multiple effects on the central nervous system [8].

Substantial preclinical evidence indicates that some flavonoids reduce behavioral endophenotypes of depression in animal models by increasing the concentrations of different neurotransmitters and expression of neurotrophic factors in the brain [9, 10]. The present review focuses on the results of preclinical research that indicate the potential antidepressant effects of some flavonoids and describes the mechanisms of action that are involved in these effects. We propose future scientific research in the area of pharmacotherapy to develop safe and effective antidepressant drugs based on natural products to ameliorate the symptoms of depression in humans.

## 2. Background on Flavonoids

**Figure 1:**
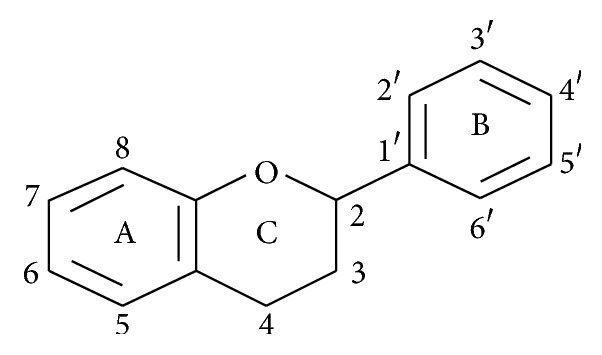
Basic structure of flavonoids and the system of numeration. A and B are phenyls, and C corresponds to pyrene. Numbers indicate the numeration system of the basic structure of flavonoids.

Flavonoids are phenolic compounds that are widely distributed in vascular plants. Many chemical compounds, both in their free form and in the form of glycosides, have been evaluated to determine their biological activity [11]. More than 5000 types of flavonoids have been identified, which are structurally different and possess a wide range of biological activities. Flavonoids are chemical compounds with a low molecular weight whose base structure (Figure 1) comprises a system of rings of diphenyl pyrene or phenyl benzopyrene accompanied by two variable groups of hydroxyl phenolic radicals [12].

**Figure 2:**
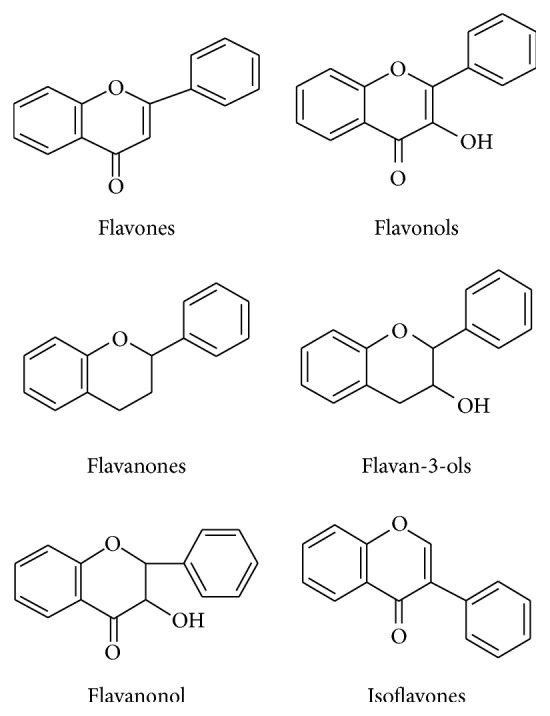
General classification and basic structure of flavonoids.

According to the chemical structure of flavonoids (Figure 2), they can be classified as flavonoids, flavones, flavanones, isoflavones, and anthocyanidins [62]. Studies in mammals and* in vitro* have shown that flavonoids exert antioxidant, antiallergic, hepatoprotective, antiviral, anticarcinogenic, neuroprotective, antitoxic, anxiolytic, antiepileptic, estrogenic, and antidepressant-like effects by inhibiting some enzymes [63–66], which is dependent on the dose and type of flavonoid administered.

## 3. Flavonoid Metabolism

The daily dietary intake of flavonoids in humans is approximately 1-2 g per day, principally depending on individual alimentary habits [67]. Most flavonoids are found in plants in the *β*-glycoside form. After intake, the processes of hydrolysis occur, but since the union-*β* in these sugars is resistant to the hydrolysis produced by pancreatic enzymes, this metabolic process occurs in the intestinal lumen through actions of the lactase phlorizin hydrolase that is located in the membrane of enterocytes. When phlorizin hydrolase hydrolyzes flavonoids, they become capable of crossing intestinal membranes through passive diffusion. Another enzyme that facilitates the hydrolysis of flavonoids is cytosolic *β*-glycosidase, which hydrolyzes a high number of glycosides [68, 69]. Cytosolic *β*-glycosidase is located intracellularly in erythrocytes; therefore, active transport is required to cross cellular membranes. It is produced by sodium-glucose transport protein, which depends on sodium (SGLT-1) [69].

Hydrolyzed flavonoids (aglycones) are conjugated through methylation, sulphatation, and glucuronidation. Because of their high conjugation, hydrolyzed flavonoids are detected in low concentrations in plasma [70]. For example, hesperetin aglycone (the active form of the flavonoid hesperidin) is metabolized by the cytochrome isoforms P450 CYP1A and CYP1B1. This first-pass metabolism principally occurs through intestinal cells [71]. The metabolites of hesperidin/hesperetin are eliminated by renal route. Their metabolites are found in urine but not feces, suggesting that the high bacterial degradation of phenolic acids occurs at the level of the colon, allowing passage to the systemic circulation [71]. Particularly, the elimination of the flavonoid chrysin depends on the outflow, in which conjugated structures are hydrolyzed through sulphatases and glucuronidases in the intestine, suggesting that chrysin has low intestinal absorption, in which it is detected in high concentrations in feces [72].

Some studies have shown that hydrolyzed flavonoids and their conjugated derivatives may cross the hematoencephalic barrier and exert actions on the central nervous system [73]. This may at least partially explain their multiple pharmacological actions at the neuronal level that affect cognition and emotional and affective states. Numerous preclinical studies have shown that some flavonoids reduce depressive-like behavior, and these effects are related to the activation of neurotransmitter systems and trophic factors in the brain.

## 4. Antidepressant-Like Effects of Flavonoids in Plant Extracts

The treatment of depressive disorders is principally based on the use of synthetic antidepressant drugs (e.g., tricyclic antidepressants, selective serotonin reuptake inhibitors, and dual-action antidepressants) that are clinically effective but produce side effects. A principal limiting factor in the use of antidepressant drugs is their delayed onset of therapeutic antidepressant effects. Generally, therapeutic effects in humans occur after 2-3 weeks of treatment through neuronal plastic changes and the modification of neurotransmitter receptors. This process requires a relatively long time to produce antidepressant effects [74, 75]. In the first weeks of antidepressant treatment, patients may experience a worse mood state compared with their state before the initiation of pharmacological treatment [76]. Patients have sought therapeutic alternatives to ameliorate symptoms of depression. Infusions or standardized extracts of plants have been used for the alternative treatment of depression [77]. However, in most cases, these alternative therapies have not been investigated in systematic studies to support or refute their purported medicinal properties. Such a dearth of studies can pose a health risk to patients. Preclinical studies have evaluated the effects of plant extracts that contain a high percentage of total flavonoids (Table 1) that produce antidepressant-like effects in animal models of depression through actions on neurotransmitter receptors and production of neurotrophic factors in the brain [40].

Behavioral models (e.g., tail suspension test, forced swim test, and chronic unpredictable mild stress [CUMS] paradigm) allow identification of the potential antidepressant effects of diverse natural substances as flavonoids [78, 79], among others. Naringenin (10, 20, and 50 mg/kg), an isoflavone isolated from citrus peel, reduced total immobility time in the tail suspension test in male mice, similar to the effects of 20 mg/kg fluoxetine, a clinically effective antidepressant drug. These effects were interpreted as potential antidepressant-like effects [52]. Interestingly, this effect was blocked by pretreatment with* p*-chlorophenylalanine methyl ester (100 mg/kg) and *α*-methyl-*p*-tyrosine (100 mg/kg), inhibitors of the synthesis of serotonin and norepinephrine, respectively [52]. This suggests that the mechanism of action of naringenin involves the activation of serotonergic and noradrenergic neurotransmitter systems in the brain. Additionally, 10 and 20 mg/kg naringenin increased the expression of brain-derived neurotrophic factor (BDNF) in the hippocampus after 21 days of treatment in mice that were subjected to CUMS [28], which was associated with an antidepressant-like effect. These results indicate that the antidepressant-like effect of naringenin may be mediated by the activation of both neurotransmitter systems and neurotrophic factors. Such mechanisms of action have also been identified for other clinically effective antidepressant drugs, such as fluoxetine [80].

Park et al. (2006) [81] found that a standardized extract of* Cirsium japonicum* Fisch. ex DC (Asteraceae) produced antidepressant-like effects in male mice. This effect was replicated in subsequent studies that evaluated the antidepressant-like effect of an ethanolic extract of this plant at doses of 50, 100, 200, and 400 mg/kg and its principal chemical constituents (i.e., linarin, pectolinarin, chlorogenic acid, and luteolin) at doses of 10 mg/kg in the forced swim and open field tests [30]. The authors showed that the antidepressant-like effects of this plant extract were produced by the flavonoid luteolin through actions on the GABA_A_ receptor. Such GABA_A_ receptor activation has also been involved in the antidepressant-like activity of other plant metabolites [82, 83] and some neurosteroids such as allopregnanolone [84–86].

In male Sprague-Dawley rats, CUMS and an acute injection of corticosterone were used to produce depression-like behavior. The antidepressant-like effects of the flavonoid icariin (60 mg/kg), isolated from* Epimedium brevicornum *Maxim (Berberidaceae) on depression-like behavior produced by CUMS or corticosterone injection, were evaluated in the forced swim test. Corticosterone and CUMS increased total immobility time, reflecting despair-like behavior, and reduced BDNF concentrations in the hippocampus. These effects were prevented by the administration of icariin flavonoid, which was associated with the antidepressant-like effect [32].

A preclinical study of the methanolic extract of* Byrsonima crassifolia* (L.) Kunth (Malpighiaceae) at a dose of 500 mg/kg reported an antidepressant-like effect that was similar to the clinically effective antidepressant imipramine in albino ICR mice in the forced swim test. The authors indicated that this antidepressant-like effect was attributable to flavonoids in the extract [21], corresponding to quercetin (1.4 mg/kg), rutin (4.4 mg/kg), and hesperidin (0.7 mg/kg), which produce antidepressant-like effects when they are individually injected [9, 54, 87, 88]. Additionally, it has been reported that the administration for 7 days of flavonoid quercetin (10, 50, and 200 mg/kg,* p.o.*) decreases the 5-hydroxyindole acetaldehyde production modulating the serotonergic system by attenuating mitochondrial MAO-A activity in the brain [89], which is involved in the therapeutic effect of some antidepressant drugs.

Oral administration of 25, 50, and 100 mg/kg of a standardized aqueous extract, referred to as* Xiaobuxin-Tang*, which contains four different natural products (i.e.,* Haematitum*,* Flos Inulae*,* Folium Phyllostachydis Henonis*, and* Semen Sojae Preparatum*), reduced immobility time in both the forced swim and the tail suspension tests in lipopolysaccharide-treated ICR mice, thus demonstrating an antidepressant-like effect.* Xiaobuxin-Tang* also reduced the levels of proinflammatory cytokines in the brain [90], apparently by its high content of flavonoids. A reduction of immobility time in the forced swim test was also produced by acute or chronic administration of 30, 100, and 300 mg/kg of aqueous [91] or ethanolic [92] extracts of* Melissa officinalis* L. (Lamiaceae). This same effect was produced by its active metabolite rosmarinic acid (36 mg/kg) in male Sprague-Dawley rats [91], and the authors suggested that the antidepressant-like effect of this extract could be associated with its high content of rosmarinic acid, which is able to modulate the serotonergic system [91]. However, it is not possible to discard the participation of other chemical constituents of the* M. officinalis* extracts in their antidepressant-like effects, considering the high content in essential oils and flavonoids such as quercitrin, apigenin, and luteolin derivatives that may inhibit monoamine oxidases A (MAO-A) activity and interact with the GABA_A_ receptors [93], which also occurs with the majority of the conventional antidepressant drugs [94].

*Glycyrrhiza uralensis* Fisch. (Fabaceae) is another plant with potential antidepressant-like effects that are associated with its content of at least five flavonoids (i.e., liquiritin, liquiritigenin, isoliquiritigenin, isoononin, and 7,4′-dihydroxyflavone). An extract of this plant inhibited the production of tumor necrosis factor-*α* (TNF-*α*) in microglial cells in mice [95]. These findings are important because TNF-*α* has been detected in high concentrations in patients with anxiety and depression symptoms. Therefore, a reduction of TNF-*α* could be beneficial for ameliorating symptoms of anxiety and depression, as is the case with other antidepressant agents. The flavonoid isoliquiritigenin also inhibits TNF-*α* and increases the concentration of BDNF in the hippocampus and cerebral cortex [95]. Administration of the flavonoid 5,7-dihydroxyflavone (chrysin) at doses of 1 and 10 mg/kg for 60 days increased BDNF concentrations in the hippocampus and prefrontal cortex [96] and produced antidepressant-like effects in the forced swim test in mice [10]. These data are relevant because higher plasma and brain concentrations of BDNF were detected when clinically effective antidepressant drugs were administered in experimental animals (for review, see [97]) and depressed patients (for review, see [80]), suggesting that flavonoids have a similar pharmacological profile as conventional antidepressant drugs.

Su et al. (2014) [98] evaluated the effects of the Chinese herbal formula* Xiao Chai Hu Tang*, which contains parts from plants described as Radix Bupleuri Chinensis, Radix Scutellariae Baicalensis, ginseng, Rhizoma Pinelliae Ternatae, Radix Glycyrrhiza Uralensis, Rhizoma Zingiberis Recens, and Fructus Jujubae. This herbal preparation contains a high percentage of flavonoids, glycosylated flavonoids, and saponins. The authors tested the effects of administration of 0.6, 1.7, and 5 mg/kg for 4 weeks. The extract was administered in male Sprague-Dawley rats subjected to CUMS, and the effects were evaluated in the open field test; glucose preference and consumption and food consumption were also evaluated. The results showed that CUMS reduced glucose preference and food consumption, reflecting anhedonia, which is a principal symptom in depressed patients. Interestingly, these deleterious effects of CUMS were prevented by the herbal preparation* Xiao Chai Hu Tang*, which was associated with higher levels of BDNF, nerve growth factor (NGF), and tropomyosin receptor kinase A (TrkA) and tropomyosin receptor kinase B (TrkB) in the hippocampus [98].

Another study explored the effects of a standardized extract used in traditional Chinese medicine. This herbal preparation,* Xiaobuxin-Tang*, includes* Flos Inulae*,* Folium Phyllostachydis Henonis*, and* Semen Sojae Preparatum* and contains a high percentage of flavonoids. A dose of 100 mg/kg of this extract produced antidepressant-like effects in male ICR mice, which were blocked by pretreatment with l-arginine (750 mg/kg), a precursor of nitric oxide synthesis. Coadministration of 7-nitroindazole (50 mg/kg), an inhibitor of nitric oxide synthesis, potentiated the action of an ineffective dose of* Xiaobuxin-Tang* (50 mg/kg) to produce antidepressant-like effects [41]. These findings suggest that the antidepressant-like effect of this extract involves nitric oxide signaling. A similar mechanism has been reported for lamotrigine, which also has antidepressant-like activity [99].

*Apocynum venetum* L. (Apocynaceae) extract produces antidepressant-like effects in male CD rats subjected to the forced swim test, apparently by their high content of hyperoside and isoquercitrin which are major flavonoids in the extract [100]. Different doses (25, 50, and 100 mg/kg) of an* Apocynum venetum* L. extract that contained a high percentage of flavonoids were also evaluated in male ICR mice [18]. The 50 and 100 mg/kg doses significantly reduced immobility time in both the forced swim test and the tail suspension test, without producing nonspecific effects on motor activity in the open field test, a typical effect of substances with antidepressant activity [101]. These antidepressant-like effects were associated with higher concentrations of norepinephrine and dopamine and their metabolites 3,4-dihydroxyphenylacetic acid (DOPAC) and homovanillic acid (HVA), respectively, in the hippocampus. Furthermore, these antidepressant-like effects were blocked by pretreatment with the dopamine D_1_ receptor antagonist SCH23390 (0.05 mg/kg) and D_2/3_ receptor antagonist sulpiride (50 mg/kg) [18], confirming that the antidepressant-like effects of* Apocynum venetum* L. occur through actions on the dopaminergic system. This mechanism of action is important because clinically effective antidepressant drugs, such as clomipramine (tricyclic antidepressant) and fluoxetine (selective serotonin reuptake inhibitor), activate the serotonergic and noradrenergic systems in the long term and parallelly also activate the mesolimbic dopamine system producing their antidepressant effects [102–104].

The aforementioned data show that flavonoids and likely other active metabolites that are contained in plant extracts may contribute to the antidepressant-like effects of plants that are used in traditional medicine to ameliorate symptoms of depression. These beneficial effects appear to occur through the activation of neurotransmitter systems and other neuronal processes. The activation of neurotrophic factors, such as BDNF, significantly impacts neuronal function. The activation of neurotransmitter systems (i.e., principally serotonergic, noradrenergic, and dopaminergic) in specific brain areas (e.g., hippocampus and prefrontal cortex) reactivates chemical communication in the long term, thus allowing plastic changes and subsequently the therapeutic effects of antidepressant drugs [105]. Preclinical research has also investigated the effects of specific flavonoids that are extracted from medicinal plants. These flavonoids have been purified, chemically characterized, and prepared for administration. Such efforts have allowed the identification of specific flavonoids that have potential antidepressant-like effects.

## 5. Antidepressant-Like Effects of Flavonoids Isolated from Plants

Flavonoids produce pharmacological actions on the central nervous system (Table 2) to regulate emotional and mood states associated with plastic and neurochemical changes as is the case with conventional antidepressant drugs [9, 10, 38, 101].

Preclinical studies have also reported the potential antidepressant-like effects of specific flavonoids (Table 3). Hesperidin is a flavonoid that has different pharmacological actions (e.g., antioxidant, antineoplastic, and neuroprotective effects)* in vitro* and* in vivo*. This flavonoid has been studied as a potential antidepressant agent because of its actions on the serotonergic, dopaminergic, and noradrenergic systems. The administration of 0.1, 0.3, and 1 mg/kg hesperidin* (i.p.)* for 21 days in Swiss mice significantly reduced total immobility time in the tail suspension test. This antidepressant-like effect was associated with a significant increase in BDNF concentrations in the hippocampus [9] and actions at the 5-HT_1A_ receptors [106]. Also, the administration of 10, 20, and 40 mg/kg astilbin* (i.p.)* for 21 days in male C57BL/6L mice exerted antidepressant-like effects in the forced swim test, tail suspension test, and CUMS paradigm, and these effects were associated with an increase in BDNF concentrations in the cerebral cortex. These effects were similar to those produced by 10 mg/kg of the tricyclic antidepressant imipramine [34].

The behavioral and molecular effects of the flavonoid baicalein (40 mg/kg,* i.p.,* for 14 days) were evaluated in male Sprague-Dawley rats. Baicalein significantly reduced total immobility time, similar to the antidepressant fluoxetine, in the forced swim test. This antidepressant-like effect was associated with activation of the dopaminergic system and greater expression of BDNF* m*RNA in the hippocampus, an effect also detected with the antidepressant fluoxetine [35]. In support, injections of baicalein (1, 2, and 4 mg/kg*, i.p.*, for 21 days) in male Kunming mice subjected to CUMS reduced immobility time in the forced swim and tail suspension tests, which was accompanied by an increase in extracellular signal-regulated kinase and BDNF expression in the hippocampus, similar to 15 mg/kg of the antidepressant imipramine [36].

Another flavonoid, baicalin, isolated from the dried root of* Scutellaria baicalensis* Georgi (Labiatae), produces an antidepressant-like effect in the forced swim and tail suspension tests in mice treated with 25 and 50 mg/kg,* p.o.* This effect was similar to that produced by 20 mg/kg of the antidepressant fluoxetine. Apparently, the baicalin effect was associated with inhibition of monoamine oxidase enzymes types A and B [107], a mechanism of action involved in the therapeutic effect of some antidepressant drugs.

The administration of 10, 20, 30 mg/kg of the flavonoid vitexin* (p.o.)* also significantly reduced total immobility time in both the forced swim and the tail suspension tests. Interestingly, animals treated with vitexin exhibited a significant increase in the time spent climbing in the forced swim test [53], suggesting that activation of the noradrenergic system may be involved in the antidepressant-like effect of this flavonoid. A selective increase in the time spent climbing is only produced by antidepressant drugs that act on the noradrenergic system [108]. Injections of the serotonin 5-HT_1A_ receptor antagonist 1-(2-methoxyphenyl)-4-(4-[2-phthalimido]butyl)-piperazine (NAN-190) or dopamine receptor antagonist SCH23390 blocked the antidepressant-like effect of vitexin [53], indicating that the antidepressant-like effects involve the activation of at least three neurotransmitter systems (i.e., serotonergic, noradrenergic, and dopaminergic). Similarly, the flavonoid nobiletin (25, 50, and 100 mg/kg,* p.o.*), isolated from citrus peels, produces antidepressant-like effects in the forced swim and tail suspension tests in male ICR mice. Interestingly, these effects are blocked by previous injection of WAY 100635 (a serotonin 5-HT_1A_ receptor antagonist), cyproheptadine (a serotonin 5-HT_2A_ receptor antagonist), prazosin (an *α*_1_-adrenoceptor antagonist), SCH23390 (a dopamine D_1_ receptor antagonist), or sulpiride (a dopamine D_2_ receptor antagonist), showing that the antidepressant-like effect of nobiletin involves participation of serotonergic, noradrenergic, and dopaminergic systems [109], as is the case as well with bioflavonoid apigenin in several brain structures [59]. This multiple mechanism of action is unsurprising. The administration of standardized herbal products or phytomedicines prepared with* Hypericum perforatum* L. (Hypericaceae) extracts activates multiple neurotransmitter systems and produces both preclinical and clinical antidepressant effects [110–112]. However, these multiple actions have been associated with some severe side effects [113]. Further studies are necessary to explore the multiple actions of flavonoids in the brain under different experimental conditions (e.g., acute or chronic treatment) to identify potential side effects to ensure consumer safety.

Other flavonoids with antioxidant, anti-inflammatory, and neuroprotective effects have also been evaluated as potential antidepressant agents, one example of which is the flavonoid fisetin. The administration of 10 and 20 mg/kg fisetin* (i.p.)* significantly reduced total immobility time in the forced swim and tail suspension tests [38]. This antidepressant-like effect was apparently produced by activation of the serotonergic system. The blockade of serotonin synthesis by pretreatment with* p*-chlorophenylalanine blocked the antidepressant-like effect of fisetin. This study also found that fisetin inhibited the activity of MAO-A, which is involved in the metabolism of serotonin and norepinephrine [38]. Similarly to other flavonoids, fisetin seems to exert its antidepressant-like effects through at least two different mechanisms of action: activating the serotonergic system and inhibiting monoamine metabolism. However, other neurotransmitter systems could be involved in the antidepressant-like effect produced by flavonoids. Two synthetic flavones, 3′-methoxy-6-methylflavone and 3′-hydroxy-6-methylflavone, in doses of 100 mg/kg,* i.p.*, produce antidepressant-like effects in the forced swim and tail suspension tests, similar to antidepressant imipramine [114]. Interestingly, the effect produced by both synthetic flavonoids was partially ameliorated by coadministration of bicuculline (a competitive *γ*-aminobutyric acid binding site antagonist), suggesting the modulation/direct activation of the GABA_A_ receptors, as is the case with neurosteroids with antidepressant-like activity [85, 86].

Depressive disorders are highly prevalent in diabetic patients. Using a preclinical model of diabetes that was induced by streptozotocin in mice, the effects of the bioflavonoid quercetin (50 and 100 mg/kg,* i.p.*) were compared with fluoxetine (5 mg/kg,* i.p.*) and imipramine (15 mg/kg,* i.p.*) in the forced swim test [115]. Results showed that quercetin significantly reduced depressive-like behavior in diabetic mice, similar to the conventional antidepressants fluoxetine and imipramine. Interestingly, the quercetin-induced reduction of depressive-like behavior was only detected in diabetic mice and not in healthy mice, while fluoxetine and imipramine produced antidepressant-like effects in both diabetic and healthy mice. In another study, quercetin (50 mg/kg,* i.p.*, for 21 days) also exerted antidepressant-like effects in diabetic rats in the forced swim test. These effects did not involve regulation of the hypothalamic-pituitary-adrenal axis, in which this flavonoid did not produce significant changes in plasma adrenocorticotropic hormone or corticosterone concentrations [54]. These data suggest that quercetin may have a mechanism of action that is different from conventional antidepressants. The antidepressant-like effects of quercetin have been suggested to primarily occur through antioxidative actions and a reduction of proinflammatory cytokine concentrations in the brain [54] that in the long term restore neurochemical function as is the case with conventional antidepressant drugs. Future studies should explore the ability of quercetin to ameliorate symptoms of depression, particularly in diabetic patients.

Finally, studies of the neurobiological bases of depressive disorders and mechanisms of action of antidepressant drugs have shown that reductions of neurotransmitter system activity and BDNF concentrations are associated with depressive symptoms in humans [116] and depression-like behavior in stressor-exposed rats [42]. A reduction of BDNF synthesis has been observed in the hippocampus and cerebral cortex, among other brain structures, in experimental animals. Antidepressant drugs increase BDNF production in both animals and depressed patients [97, 117], suggesting a negative correlation between BDNF concentrations and the severity of depressive symptoms.

Mice that are subjected to CUMS develop symptoms of anhedonia (e.g., a reduction of sucrose preference and consumption) and depressive-like behavior (e.g., increase in immobility time in the forced swim test), and these effects were prevented by oral administration of 5 and 20 mg/kg of the flavonoid chrysin after 28 days of treatment. This antidepressant-like effect of chrysin was accompanied by an increase in BDNF concentrations in the hippocampus and prefrontal cortex and the activation of NGF in mice [10]. Additionally, flavonoid chrysin (5 and 20 mg/kg,* p.o.*, 28 days), similar to antidepressant fluoxetine (10 mg/kg,* p.o.*, 28 days), increases serotonin concentration and reduces the indoleamine-2,3-dioxygenase and caspases 3 and 9 activities in the prefrontal cortex and hippocampus in C57B/6J mice subjected to CUMS, which was associated with the antidepressant-like effect detected in the tail suspension test [118], with the participation of BDNF. Similarly, the administration of 20 and 40 mg/kg of the flavonoid orientin for 21 days also produced antidepressant-like effects in mice that were subjected to CUMS, and this effect was associated with the activation of BDNF and an increase in serotonin and norepinephrine concentration in the hippocampus and cerebral cortex [40]. The administration of 20 and 40 mg/kg of the flavonoid icariin for 35 days also produced antidepressant-like effects in rats that were subjected to CUMS. In that study, control animals presented significant neuronal damage and neuroinflammation in the hippocampus, which were associated with higher oxidative stress. These deleterious effects were reversed by the administration of icariin at doses that reduced depressive-like behavior [42]. These studies suggest that the antioxidant activity and the activation of monoaminergic systems are associated with the production of BDNF by flavonoids [119], ultimately producing antidepressant-like effects in animals. However, this hypothesis requires further exploration.

## 6. Concluding Remarks

Preclinical data on the antidepressant-like effects of some flavonoids have consistently reported behavioral effects and neurochemical actions in the brain, thus supporting the potential therapeutic application of these natural compounds for the amelioration of depressive symptoms in humans. The data that were reviewed herein implicate BDNF in the antidepressant-like effects of flavonoids. This mechanism of action is relevant because it has been associated with the actions of clinically effective antidepressant drugs [80, 120]. BDNF modulates neurotransmitters and receptor activity and is involved in the activation of serotonergic, noradrenergic, and dopaminergic pathways and neurogenesis in the hippocampus and cerebral cortex, which are implicated in the neurobiology of psychiatric disorders, including depression.

Activation of BDNF and TrkB is produced after administration of conventional antidepressant drugs, such as fluoxetine and citalopram [28, 101, 121], which is associated with the reduction of most of the symptoms of depression [97, 122–124]. Some flavonoids (e.g., 7,8-dihydroxyflavone) also act as TrkB receptor agonists and stimulate neurogenesis in the hippocampus [41]. Such findings may reveal new possibilities for the development of therapeutic alternatives for the treatment of depression, including the administration of subthreshold doses of flavonoids combined with conventional antidepressant drugs. Combined administration of both substances could likely produce antidepressant-like effects with a shorter onset of action through the early stimulation of BDNF production and parallelly modify the neurotransmitter receptor function, which requires further exploration.

Finally, despite the positive findings regarding the antidepressant-like effects of some flavonoids at the preclinical level, potential side effects of long-term consumption need to be investigated, including studies of toxicology and possible pharmacological interactions with other substances, to determine the tolerability and safety of flavonoids in humans. Such studies may eventually demonstrate that some flavonoids are safe alternatives for the treatment of depressive disorders in clinical practice.

## Figures and Tables

**Table 1:** Plants with antidepressant-like effects associated with their total flavonoids content.

Plant (family)	Doses (animal)	Duration of treatment	Behavioral test	Reference
*Alpinia oxyphylla *Miq. (Zingiberaceae)	10 mg/kg, *p.o.* (A)	14 days	FST, SPT	[13]
*Hemerocallis citrina *L. (Xanthorrhoeaceae)	400 mg/kg, *p.o.* (B)	Single dose	TST	[14]
	10, 20, and 40 mg/kg, *p.o.* (C)	35 days	SPT	[15]
*Apocynum venetum *Linn. (Apocynaceae)	0.35 mM/kg, *i.p.* (B)	Single dose	FST, TST	[16]
*Hibiscus esculentus *L. (Malvaceae)	500 and 750 mg/kg, *i.p.* (D)	Single dose	FST, TST	[17]
*Apocynum venetum* L. (Apocynaceae)	50 and 100 mg/kg, *p.o.* (E)	10 days	FST, TST	[18]
*Glycyrrhiza uralensis *Fisch. (Fabaceae)	30, 100, and 300 mg/kg, *p.o.* (F)	28 days	FST, TST	[19, 20]
*Byrsonima crassifolia* (L.) Kunth (Malpighiaceae)	500 mg/kg, *p.o.* (E)	Single dose	FST	[21]
*Cecropia pachystachya* Trécul (Urticaceae)	50 mg/kg, *p.o.* (G)	8 days	FST	[22]
*Chrysactinia mexicana *A. Gray (Asteraceae)	1, 5, 10, 100, and 200 mg/kg, *p.o.* (H)	Single dose	FST	[23]
*Opuntia ficus-indica* (L.) Mill. (Cactaceae)	30 mg/kg, *p.o.* (E)	14 days	FST, TST	[24]
*Hibiscus rosa-sinensis* Linn. (Malvaceae)	30 and 100 mg/kg, *p.o.* (C)	Single dose	FST, TST	[25]
*Actaea spicata *L. (Ranunculaceae)	200 mg/kg, *p.o.* (I)	Single dose	FST	[26]
*Clerodendrum serratum* Linn. (Verbenaceae)	25 and 50 mg/kg, *p.o.*	7 days	FST, TST	[27]

(A) Male Kunming mice; (B) male mice; (C) male Sprague-Dawley rats; (D) male Swiss albino mice; (E) male ICR mice; (F) rats; (G) male Wistar rats; (H) male Swiss Webster mice; (I) male LACA mice. FST: forced swim test; TST: tail suspension test; SPT: sucrose preference test.

**Table 2:** Neurobiological effects produced by some flavonoids.

Flavonoid	Doses	Treatment duration	Effects	Reference
Naringenin	5, 10, and 20 mg/kg	21 days	Increase in BDNF concentrations in the hippocampus in male mice	[28]
	5, 10, and 20 mg/kg	14 days	Increase in 5-HT, DA, and NE in the hippocampus in male ICR mice	[29]
Luteolin	10 mg/kg	30 min before test	Increases in chloride ion flow at the GABA_A_ receptor in male rats	[30]
	50 mg/kg	23 days	Attenuation of the expression of endoplasmic reticulum stress-related proteins in the hippocampus in male ICR mice	[31]
Icariin	60 mg/kg	21 days	Increases in BDNF concentrations in the hippocampus in male rats	[32]
Hesperidin	0.01, 0.1, 0.3, and 1 mg/kg	21 days	Increase in BDNF concentrations in the hippocampus in male mice	[9]
	50 mg/kg	13 days	Increase in BDNF and NGF concentrations in the hippocampus in male C57BL/6 mice	[33]
Astilbin	10, 20, and 40 mg/kg	21 days	Increase in BDNF concentrations in the cerebral cortex in male mice, similar to imipramine	[34]
Baicalein	10, 20, and 40 mg/kg	14 days	Increase in dopamine and BDNF concentrations in the hippocampus in male rats	[35]
	1 and 4 mg/kg	Single injection or 21 days	Restoring of the reduction of extracellular signal-regulated kinase phosphorylation and BDNF expression in the hippocampus of male Kunming mice subjected to CUMS	[36]
Chrysin	5 and 20 mg/kg	28 days	Increase in BDNF concentrations in the hippocampus and prefrontal cortex in female mice	[10]
	5 and 20 mg/kg	14 days	Increase in 5-HT and BDNF concentrations in the hippocampus in male C57B/6J mice	[37]
Fisetin	5, 10, and 20 mg/kg	60 min before test	Activation of the serotonergic system, apparently through inactivation of MAO-A enzyme in male mice	[38]
	5 mg/kg	14 days	Increases in phosphorylated TrkB (pTrkB) in the hippocampus in male ICR mice	[39]
Orientin	20 and 40 mg/kg	21 days	Increase in BDNF, serotonin, and norepinephrine concentrations in the hippocampus and prefrontal cortex in male mice	[40]
7,8-Dihydroxyflavone	1, 3, and 10 mg/kg	60 min before test	Increase in BDNF concentrations in the hippocampus and prefrontal cortex in male mice	[41]
Icariin	20 and 40 mg/kg	35 days	Decrease in oxidative stress and neuroinflammation in the hippocampus in male rats	[42]
Dihydromyricetin	10 and 20 mg/kg	7 days	Increase in *m*RNA for BDNF in the hippocampus in male C57BL/6 mice	[43]
Silymarin	100 and 200 mg/kg	14 days	Increase in 5-HT, DA, NE, and BDNF concentration in the hippocampus and cerebral cortex, similar to fluoxetine in adult Wistar rats	[44]
Myricitrin	10 mg/kg	21 days	Increases in cell proliferation in the subgranular zone of the hippocampal dentate gyrus in male BALB/c mice	[45]
Myricetin	50 mg/kg	21 days	Increases in BDNF concentrations in the hippocampus in male C57BL/6 mice	[46]
3,5,6,7,8,3′,4′-Heptamethoxyflavone	50 and 100 mg/kg	15 days	Increase in BDNF concentration, neurogenesis, and neuroplasticity in the hippocampus in male C57BL/6 mice	[47, 48]
Apigenin	20 and 40 mg/kg	21 days	Increase in BDNF concentrations in the hippocampus in male ICR mice	[49]
Miquelianin	0.6 mg/kg	14 days	Modulation of the hypothalamic-pituitary-adrenal axis by reducing plasma concentration of ACTH and corticosterone in male CD rats	[50]
Isoquercitrin	0.6 mg/kg	14–56 days	Modulation of the hypothalamic-pituitary-adrenal axis by reducing plasma concentration of ACTH and corticosterone in male CD rats	[50]
Liquiritin and isoliquiritin	20 mg/kg	30 min before sample	Increases in 5-HT and NE concentrations in the hippocampus, hypothalamus, and cortex in mice	[51]

BDNF: brain-derived neurotrophic factor; NGF: nerve growth factor; MAO-A: monoamine oxidase type A; TrkB: tropomyosin receptor kinase B; 5-HT: serotonin; DA: dopamine; NE: norepinephrine; ACTH: adrenocorticotropic hormone.

**Table 3:** Effect of flavonoids on depression-like behavior at preclinical research.

Model of depression	Flavonoid (animal)	Doses	Treatment duration	Effect	Reference
Forced swim test^1^	Naringenin (A)	10, 20, and 50 mg/kg, *p.o.*	60 min before test	No effect	[52]
	Luteolin (A)	10 mg/kg, *p.o.*	30 min before test	Antidepressant	[30]
		50 mg/kg, *p.o.*	23 days	Antidepressant	[31]
	Icariin (B)	60 mg/kg, *p.o.*	21 days	Antidepressant	[32]
		20 and 40 mg/kg, *p.o.*	35 days	Antidepressant	[42]
	Astilbin (C)	10, 20, and 40 mg/kg, *i.p.*	21 days	Antidepressant	[34]
	Baicalein (B)	10, 20, and 40 mg/kg, *i.p.*	14 days	Antidepressant	[35]
	Baicalein (F)	1, 2, and 4 mg/kg, *i.p.*	Single injection or 21 days	Antidepressant	[36]
	Kaempferol (A)	30 mg/kg, *p.o.*	14 days	Antidepressant	[24]
	Quercitrin (A)	30 mg/kg, *p.o.*	14 days	Antidepressant	[24]
	Vitexin (D)	10, 20, and 30 mg/kg, *p.o.*	60 min before test	Antidepressant	[53]
	Chrysin (J)	5 and 20 mg/kg, *p.o.*	28 days	Antidepressant	[10]
	Fisetin (A)	5, 10, and 20 mg/kg, *p.o.*	60 min before test	Antidepressant	[38]
	Quercetin (E)	50 and 100 mg/kg, *i.p.*	21 days	Antidepressant	[54]
		40 and 80 mg/kg, *p.o.*	14 days	Antidepressant	[55]
	Quercetin (I)	50 mg/kg, *i.p.*	21 days	Antidepressant	[54]
	Quercetin (L)	25 and 50 mg/kg, *p.o.*	14 days	Antidepressant	[56]
	Orientin (F)	20 and 40 mg/kg, *p.o.*	21 days	Antidepressant	[40]
	7,8-Dihydroxyflavone (G)	1, 3, and 10 mg/kg, *i.p.*	60 min before test	Antidepressant	[41]
	Isosakuranetin-5-*O*-rutinoside (A)	15 and 30 mg/kg, *p.o.*	21, 18, and 1 h before test	Antidepressant	[57]
	Liquiritin (K)	10, 20, and 40 mg/kg, *p.o.*	30 min before test	Antidepressant	[51]
	Isoliquiritin (K)	10, 20, and 40 mg/kg, *p.o.*	30 min before test	Antidepressant	[51]
	Naringin (E)	50 and 100 mg/kg, *i.p.*	14 days	Antidepressant	[58]

Tail suspension test^1^	Naringenin (A)	10, 20, and 50 mg/kg, *p.o.*	60 min before test	Antidepressant	[52]
		5, 10, and 20 mg/kg, *p.o.*	14 days	Antidepressant	[29]
	Hesperidin (H)	0.1, 0.3, and 1 mg/kg, *i.p.*	21 days	Antidepressant	[9]
	Astilbin (G)	10, 20, and 40 mg/kg, *i.p.*	21 days	Antidepressant	[34]
	Vitexin (D)	10, 20, and 30 mg/kg, *p.o.*	60 min before test	Antidepressant	[53]
	Fisetin (A)	5, 10, and 20 mg/kg, *p.o.*	60 min before test	Antidepressant	[38]
	Orientin (F)	20 and 40 mg/kg, *p.o.*	21 days	Antidepressant	[40]
	7,8-Dihydroxyflavone (G)	3 and 10 mg/kg, *i.p.*	60 min before test	Antidepressant	[41]
	Baicalein (F)	1, 2, and 4 mg/kg, *i.p.*	Single injection or 21 days	Antidepressant	[36]
	Kaempferol (A)	30 mg/kg, *p.o.*	14 days	Antidepressant	[24]
	Quercitrin (A)	30 mg/kg, *p.o.*	14 days	Antidepressant	[24]
	Liquiritin (K)	10, 20, and 40 mg/kg, *p.o.*	30 min before test	Antidepressant	[51]
	Isoliquiritin (K)	10, 20, and 40 mg/kg, *p.o.*	30 min before test	Antidepressant	[51]

CUMS-sucrose intake^2^	Naringenin (A)	10 and 20 mg/kg, *p.o.*	21 days	Antidepressant	[28]
	Icariin (B)	60 mg/kg, *p.o.*	21 days	Antidepressant	[32]
		20 and 40 mg/kg, *p.o.*	35 days	Antidepressant	[42]
	Astilbin (C)	10, 20, and 40 mg/kg, *i.p.*	21 days	Antidepressant	[34]
	Chrysin (J)	5 and 20 mg/kg, *p.o.*	28 days	Antidepressant	[10]
	Orientin (F)	20 and 40 mg/kg, *p.o.*	21 days	Antidepressant	[40]
	Apigenin (A)	7 and 14 mg/kg, *p.o.*	49 days	Antidepressant	[59]
	7,8-Dihydroxyflavone (G)	10 and 20 mg/kg, *i.p.*	28 days	Antidepressant	[60]
	Icariin (B)	20 and 40 mg/kg, *p.o.*	35 days	Antidepressant	[61]

^1^The antidepressant-like effect is suggested by the reduction in immobility time without significant changes in the general locomotor activity. ^2^The antidepressant-like effect is suggested by the increase in sucrose intake. CUMS: chronic unpredictable mild stress. (A) Adult male ICR mice. (B) Male Sprague-Dawley rats. (C) Male C57BL/6J mice. (D) Adult male BALB/c mice. (E) Adult male Wistar rats. (F) Adult male Kunming mice. (G) Adult male C57BL/6 mice. (H) Adult male Swiss mice. (I) Male 21-day streptozotocin-induced diabetic Wistar rats. (J) Female C57BL/6J mice. (K) Mice sex and strain were not identified. (L) Female Swiss mice.
